# Perceptions, Expectations, and Experience of Physicians About Pharmacists and Pharmaceutical Care Services in Pakistan: Findings and Implications

**DOI:** 10.3389/fphar.2021.650137

**Published:** 2021-05-14

**Authors:** Khezar Hayat, Zia Ul Mustafa, Brain Godman, Muhammad Arshed, Jiaxing Zhang, Faiz Ullah Khan, Fahad Saleem, Krizzia Lambojon, Pengchao Li, Zhitong Feng, Yu Fang

**Affiliations:** ^1^ Department of Pharmacy Administration and Clinical Pharmacy, School of Pharmacy, Xi’an Jiaotong University, Xi’an, China; ^2^ Center for Drug Safety and Policy Research, Xi’an Jiaotong University, Xi’an, China; ^3^ Shaanxi Centre for Health Reform and Development Research, Xi’an, China; ^4^ Institute of Pharmaceutical Sciences, University of Veterinary and Animal Sciences, Lahore, Pakistan; ^5^ Department of Pharmacy Services, District Headquarter (DHQ) Hospital Pakpattan, Pakpattan, Pakistan; ^6^ Strathclyde Institute of Pharmacy and Biomedical Sciences, University of Strathclyde, Glasgow, United Kingdom; ^7^ Division of Public Health Pharmacy and Management, School of Pharmacy, Sefako Makgatho Health Sciences University, Pretoria, South Africa; ^8^ School of Pharmaceutical Sciences, Universiti Sains Malaysia, Penang, Malaysia; ^9^ Department of Emergency Medicine, Lifeline Hospital, Lahore, Pakistan; ^10^ Department of Community Health, Faculty of Medicine and Health Sciences, Universiti Putra Malaysia (UPM), Selangor Darul Ehsan, Malaysia; ^11^ Department of Pharmacy, Guizhou Provincial People’s Hospital, Guiyang, China; ^12^ Department of Pharmacy Practice, Faculty of Pharmacy & Health Sciences, University of Balochistan, Quetta, Pakistan

**Keywords:** pharmacist, physicians, perception, expectation, experience, Pakistan, interprofessional collaboration, pharmaceutical care (PC)

## Abstract

**Background:** Optimal collaboration between pharmacists and other healthcare professionals such as physicians is integral in implementing pharmaceutical care. However, there are concerns regarding the role of pharmacists, especially among low- and middle-income countries. This study explored the perceptions, expectations, and experience of physicians working in various hospital settings of Punjab, Pakistan, about pharmacists and their roles.

**Methods:** A self-administered questionnaire consisting of four sections was administered from October to December 2020. Descriptive and inferential statistics such as Kruskal-Wallis and Mann-Whitney tests were used for data analysis using SPSS.

**Results:** Six hundred and seventy-eight physicians participated in this study with a response rate of 77.9%. Most of the physicians reported minimal to no interaction with pharmacists (*n* = 521, 76.8%). However, more than three-quarters of physicians (*n* = 660, 97.3%) accepted pharmacists as evidence-based sources of drug information. In addition, many physicians (*n* = 574, 84.7%) strongly agreed that pharmacists should attend patient care rounds to respond promptly to questions related to patient medication. A limited number of physicians (*n* = 124, 18.3%) assumed that pharmacists were advising their patients regarding the judicial use of their drugs. Median expectation and experience score had a significant association with age, experience, and education of physicians (*P* < 0.05).

**Conclusions:** The perception of physicians was positive toward certain roles of pharmacists, coupled with high expectations. However, their experience was low, with most of the activities of pharmacists due to inadequate interprofessional coordination.

## Introduction

Interprofessional education and collaboration are an essential pillar of healthcare services that could significantly improve patient outcomes (Hojat and Gonnella, 2011; Schot et al., 2020). An example is the “Wise List” of recommended medicines in Stockholm County Council, Sweden, where Expert Committees comprising specialist physicians, clinical pharmacologists, and pharmacists recommend first- and second-line treatments for over 90% of the needs in ambulatory care (Gustafsson et al., 2011). Published studies have shown that high adherence rates to a recommended list of approximately 250 medicines, including hospital out-patients, have been attained in practice. Key factors to achieve high adherence rates include robust systems for selection of recommended medicines, active discussion and justification of the medicines selected in annual meetings including all key stakeholders, and comprehensive dissemination of the final “Wise List” through various electronic and other media. Few other factors that have improved the adherence rate are monitoring physician prescribing practices with active feedback and encouraging them to write an annual quality report on potential areas they will concentrate on to improve future prescribing practices (Wettermark et al., 2009; Björkhem-Bergman et al., 2013; Eriksen et al., 2017). Such comprehensive activities improve the selection of medicines for treatment and patient outcomes, with increased familiarity with the medicines prescribed reducing the potential for under-dosing, over-dosing and adverse drug reactions. Traditionally, physicians diagnose diseases and prescribe medicines while pharmacists are mainly involved in dispensing and compounding medicines. However, this is changing for several reasons. These include the exponential growth of medical and pharmaceutical sciences, an appreciable increase in drug interactions especially with a growing elderly population with more co-morbidities, and the rapidly growing cost of medicines, demanding and compelling cooperation between pharmacists and physicians (Hojat and Gonnella, 2011; Yon et al., 2020). Besides, pharmacists are a key component of healthcare services, especially in lower- and middle-income countries (LMICs) where patients may have difficulties affording both a physician and their medicines with often catastrophic consequences on family members when patients become ill (Cameron et al., 2009; Aregbeshola and Khan, 2018; Godman et al., 2020a; Haque et al., 2020). Pharmacists can also help in critical areas such as medication taking and adherence in ambulatory care, especially where health literacy is an issue, treat minor ailments and enhance appropriate prescribing and dispensing of antibiotics for infections such as upper respiratory tract infections (Abdulsalim et al., 2018; Rampamba et al., 2019; Godman et al., 2020a; Godman et al., 2020b; Ogunleye et al., 2020; Selvaraj et al., 2020). Besides, pharmacists in both hospitals and the community can promote pharmacovigilance activities, which is a concern in LMICs (Terblanche et al., 2017; Haines et al., 2020), as well as have a vital role as members of Drug and Therapeutic Committees (DTCs) guiding physicians and suggesting alternative treatments when there are shortages of medicines (Matlala et al., 2017; Terblanche et al., 2018; Matlala et al., 2020; Modisakeng et al., 2020). Consequently, this traditional relationship between physicians and pharmacists needs re-assessing to enhance the effectiveness, safety, and adherence to medicines prescribed and dispensed. In view of this, increasingly interdisciplinary teamwork is necessary to address diverse and dynamic drug and disease-related issues, in which pharmacists can play an increasing role in making a pivotal contribution to the treatment of patients (Albassam et al., 2020; Hudd, 2020).

Significant changes have been noted in the roles and responsibilities of pharmacists due to advancements in pharmaceutical services, and they are keen to adopt a critical role in managing the medication of patients, including key issues such as medication taking and adherence (Hudd, 2020; Norton et al., 2020). However, this adaptation to patient-centered pharmaceutical care can still be challenging in LMICs. This is changing although a number of key issues still need addressing. These include a chronic shortage of qualified pharmacists especially clinical pharmacists, absence of treatment guidelines in many facilities, lack of appropriate legislation promoting DTCs and other activities, and poor perception of the pharmacist as a healthcare counselor (Khan, 2011; Al-Worafi, 2014; Fakeye et al., 2017). This compares with pharmacists in Canada who are actively involved with physicians developing medication plans for patients (Long et al., 2018). In Australia, pharmacists are also providing consultative services to patients upon referral from their physicians (Tan et al., 2014), and in the United States of America, pharmacists are part of multidisciplinary teams in intensive care units enhancing patient outcomes (Preslaski et al., 2013). This is changing as seen in South Africa with their increasing role in DTC and other activities (Matlala et al., 2017; Terblanche et al., 2018; Matlala et al., 2020; Modisakeng et al., 2020) and in Kenya where hospital pharmacists are introducing new practices to improve the administration of oncology medicines (Kurgat et al., 2020).

According to the World Health Organization (WHO), the quality of pharmaceutical care offered to patients is principally dependent on collaboration and interaction between various healthcare professionals such as physicians, pharmacists, nurses, and other allied staff (WHO, 2010). However, we are aware that the perception of healthcare workers about the evolving roles of the pharmacist could differ from institution to institution. For example, in Iceland, primary care physicians do not consider pharmacists as part of the healthcare team (Blondal et al., 2017). In rural Sweden, physicians are unsure about the knowledge and clinical skills of clinical pharmacists (Sjölander et al., 2017) contrasting with their key role in the development and dissemination of the “Wise List” in Stockholm County Council and subsequent monitoring of adherence rates (Gustafsson et al., 2011). Likewise, medical students in the United States of America did not perceive that pharmacists have any role in physical examination and patient screening (Wolfe et al., 2018). Such perceptions about the role of pharmacists could act as a barrier in the provision of pharmaceutical care services, which can be overcome by effective communication and involvement (Sim et al., 2020).

Pakistan is a developing country located in the South Asian region, with a population exceeding 212.2 million (Pakistan Bureau of Statistics, 2017). Pakistan’s health-care system mainly comprises state-owned and private hospitals. State-owned healthcare is delivered through a three-tiered system, including primary, secondary, and tertiary healthcare settings. Basic Health Units (BHUs) and Rural Health Centers (RHCs) are primary health-care settings. Secondary care includes health facilities that provide inpatient, ambulatory, and acute care via Tehsil Headquarter Hospitals (THQs) and District Headquarter Hospitals (DHQs), whereas tertiary care is offered through teaching hospitals (Organization, 2020).

Currently, pharmaceutical care services in collaboration with pharmacists and physicians are limited in Pakistan, and most pharmacists are performing traditional roles such as record-keeping (Khan et al., 2020a). However, studies have highlighted the importance of cooperation between pharmacists and physicians to provide the least expensive and most effective pharmacotherapy services (Perraudin et al., 2013; Alipour et al., 2018). This collaboration though depends on physician perceptions and expectations from pharmacists, which could differ from one healthcare facility to another (Kabba et al., 2020). Consequently, we sought to elucidate the perceptions, expectations, and experience of physicians toward pharmacists and their pharmaceutical care services among secondary and tertiary hospitals of Punjab, Pakistan. We started in hospitals in view of the critical role for institutions such as DTCs in improving prescribing in hospitals through joint activities; however, we are aware that DTCs can be very variable within LMICs and that there can be extensive activities by pharmaceutical companies influencing prescribing (Fadare et al., 2018a; Fadare et al., 2018b; Ogunleye et al., 2019). We are also aware that there can be considerable concerns with the prescribing of medicines in hospitals in Pakistan (Saleem et al., 2019; Khan et al., 2020b). We chose Punjab for this initial study as it is the most populous province in Pakistan. We believe our findings could help in the development and tailoring of initiatives to optimize the collaboration among healthcare professionals including physicians and pharmacists in Punjab and wider in Pakistan to benefit patients.

## Methods

### Study Design

We undertook a cross-sectional study as we believed this was the most appropriate methodology to address this key issue (Sedgwick, 2014). Punjab currently has nine administrative divisions and 34 districts. In 2019, there were 389 hospitals, 1286 dispensaries, and 284 maternity and child welfare centers, with a total bed capacity of 60387 (Pakistan Bureau of Statistics, 2019).

### Study Tool

A systematic literature review of similar studies was undertaken to conceptualize the study tool (Alkhateeb et al., 2009; Tahaineh et al., 2009; Alipour et al., 2018; Kabba et al., 2020). An expert panel of a multidisciplinary team determined the content and face validity of the initial version of the tool. A limited number of changes were subsequently made to the tool after the initial feedback and opinion of experts. The approved version of the tool had five sections with 44-items (see Supplementary Appendix S1). The first section contained information about the participants’ demographics (age, gender, experience, education, current position, and area of practice) and the hospitals’ characteristics. The second section contained information regarding the frequency of physician-pharmacist interactions and the reasons for this interaction. The third section had fifteen questions about what physicians (defined as a professional who is licensed from Pakistan Medical Commission (PMC) to practice medicine, especially one who focuses on the diagnosis and medical care including surgery) perceive about pharmacists and their pharmaceutical care-related activities. The questions were subsequently rated on a five-point Likert scale (Strongly agree to strongly disagree), with this scale frequently used in such studies (Sabblah et al., 2017; Garcia et al., 2019). There were eleven questions in the fourth section that researched the expectations of physicians about pharmacists. Each question again had five options ranging from strongly agree to strongly disagree. In the last section, information about the experience of physicians with pharmacists and their clinical services was gathered by asking eight questions again using a five-point Likert scale.

The study tool was piloted with a small number of physicians (data excluded) to measure internal consistency. The value of Cronbach’s alpha was higher than seven, which was in the acceptable range.

### Sampling

There were 1,25,734 physicians registered with PMC from the Punjab province working in various hospital settings (Commission, 2020). The sample size of the current study was 661 calculated through Raosoft (online sample size calculator) by considering a 5% margin of error, 99% confidence interval, and 50% response distribution. A total of 870 study participants were approached by convenience and snowball sampling techniques. These included physicians working in both Private Hospitals as well as State (Government) Hospitals. Medicines are currently provided free-of-charge in government hospitals, whereas patients need to cover the costs of medicines themselves in private hospitals.

### Data Collection

A team of trained data collectors comprising hospital pharmacists and physicians was used to collect the data. Due to the COVID-19 outbreak across the country, we adopted an online method for collecting data from the participants. A Google-based online questionnaire was developed, and the link to this questionnaire was disseminated to the participants. The participants were able to record their responses after clicking the link. On the first page, information about the objective of the study, eligibility criteria, confidentiality, right to withdraw, voluntary participation, and consent were provided. The participants were encouraged to share an online link to the questionnaire with their fellow physicians. Participants who were physicians and currently working in Punjab province were included in this study. However, medical students and interns were excluded.

### Ethics

The ethics permission was obtained from the Biomedical Ethics Committee of Xi’an Jiaotong University (Ref: 2020–1,340). All participants provided consent to participate in this study.

### Statistical Analysis

The data were presented as frequency and percentages using descriptive statistics, and its normality was determined by Kolmogorov–Smirnov and Shapiro–Wilk tests. Due to the non-normal distribution of data, median and interquartile ranges (IQRs) were measured. The median perception score, median expectation score, and median experience score of the study participants were computed. Kruskal-Wallis and Mann-Whitney tests were employed on continuous data, which later compared with demographic variables. To evaluate the difference in intergroup variables, post-hoc analysis (Bonferroni correction) was also performed. All data analysis was carried out using the Statistical Package for the Social Sciences (SPSS Inc., version 18, IBM, Chicago, IL, United States). A *p* < 0.05 was accepted as statistical significance.

## Results

### Demographics

There was a total of 678 replies, giving a response rate of 77.9%, and 371 (54.7%) responding physicians were male. Most of the physicians (*n* = 436, 64.3%) had a bachelor degree in medicine and were aged 31–40 years (*n* = 248, 36.6%) with a total experience of more than 8 years (*n* = 403, 59.4%). Internal medicine (*n* = 195, 28.8%) was the most common area of practice of physicians followed by pediatrics (*n* = 172, 25.4%) and surgery (*n* = 124, 18.3%). More than three-quarters of physicians (*n* = 619, 91.3%) were prescribing more than 100 prescriptions a week (Table 1).

**TABLE 1:** Demographic information of participants (*n* = 678).

Variable	Frequency (*n*)	Percentage (%)
Gender		
Male	371	54.7
Female	307	45.3
Age (years)		
21–30	201	29.6
31–40	248	36.6
41–50	163	24.0
>50	66	9.7
Total-experience		
<1 year	41	6.0
1–5 years	209	30.8
6–10 years	28	4.1
>10 years	400	59.0
Experience in current hospital		
<1 year	60	8.8
1–4 years	193	28.5
5–8 years	22	3.2
>8 years	403	59.4
Education		
Bachelor such as MBBS	436	64.3
Postgraduation	242	35.7
Area of expertize		
Internal Medicine	195	28.8
Pediatric	172	25.4
Surgery	124	18.3
Obstetrics and Gynecology	120	17.7
Others	67	9.9
Prescription filling/week		
1–20	7	1.0
21–50	12	1.8
51–100	40	5.9
>100	619	91.3
Physician—pharmacist interaction		
Never or rarely	246	36.3
1–2 times every month	275	40.6
3–6 times every month	117	17.3
7–10 times every month	20	2.9
>10 times every month	20	2.9
Hospital type		
Tertiary care	296	43.7
Secondary care	382	56.3
Hospital ownership		
Public	512	75.5
Private	166	24.5

### Physician-Pharmacist Interaction

**FIGURE 1:**
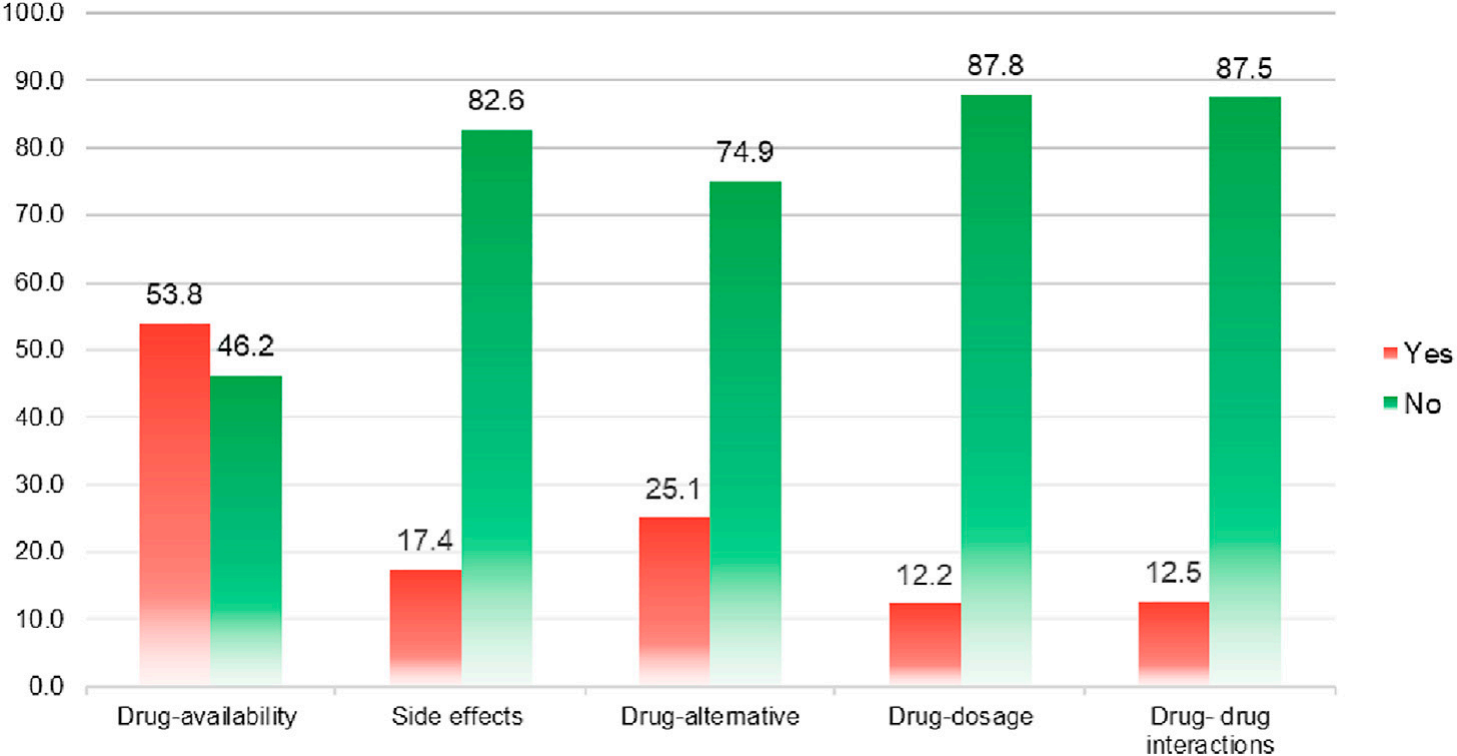
Reasons of physician-pharmacist interaction.

More than two-quarters of the physicians stated minimal to no interaction with hospital pharmacists (*n* = 521, 76.8%). Where contacted, most of the physicians were interacting with pharmacists to obtain information about drug availability (53.8%), followed by drug alternatives (25.1%) and drug side effects (17.4%) (Figure 1).

### Perception of Physicians About Pharmacists

More than three-quarters of physicians agreed that pharmacists provide evidence-based drug information (*n* = 660, 97.3%), rectify drug-related issues (*n* = 666, 98.2%) and contribute to improving patient care (*n* = 653, 96.3%). Many physicians (*n* = 627, 92.5%) believed that pharmacists could provide counseling to patients on rational drug use and play a pivotal role in enhancing patients’ outcomes (*n* = 520, 76.7%). Likewise, more than half of the physicians did not doubt their clinical knowledge (*n* = 444, 65.5%) and pharmacists’ involvement in patient care (*n* = 436, 64.3%). However, only a few physicians strongly agreed that pharmacists participate in research (*n* = 156, 23.0%) and vaccination programs (*n* = 68, 10.0%). Most physicians strongly disagreed that pharmacists are a key player in the health system (*n* = 277, 40.9%). The detailed information view of physicians about pharmacists is summarized in Table 2.

**TABLE 2:** Perception of physicians about pharmacists.

Question	Strongly agree; Strongly disagree. *N* (%)					Median (IQR)
	SA	A	N	D	SD	
They provide evidence-based drug information	475 (70.1)	185 (27.3)	16 (2.4)	1 (0.1)	1 (0.1)	1.00 (1.00)
They solve drug-related problems	479 (70.6)	187 (27.6)	11 (1.6)	1 (0.1)	0 (0.0)	1.00 (1.00)
Pharmacist contributing to improving patient care	471 (69.5)	182 (26.8)	21 (3.1)	4 (0.6)	0 (0.0)	1.00 (1.00)
They provide patient counseling on appropriate drug use	431 (63.6)	196 (28.9)	47 (6.9)	3 (0.4)	1 (0.1)	1.00 (1.00)
Pharmacists are accessible in the hospital	305 (45.0)	191 (28.2)	157 (23.2)	16 (2.4)	9 (1.3)	2.00 (2.00)
They play an important role in improving patient outcomes	307 (45.3)	213 (31.4)	149 (22.0)	1 (0.1)	8 (1.2)	2.00 (1.00)
They recommend dosage adjustments	291 (42.9)	189 (27.9)	175 (25.8)	7 (1.0)	16 (2.4)	2.00 (2.00)
They identify and help resolve adverse drug reactions	236 (34.8)	220 (32.4)	101 (14.9)	7 (1.0)	114 (16.8)	2.00 (2.00)
They take part in patient-care rounds	188 (27.7)	209 (30.8)	135 (19.9)	11 (1.6)	135 (19.9)	2.00 (2.00)
They participate in research programs	156 (23.0)	182 (26.8)	175 (25.8)	7 (1.0)	158 (23.3)	3.00 (1.00)
They participate in vaccination programs	68 (10.0)	122 (18.0)	210 (31.0)	32 (4.7)	246 (36.3)	3.00 (3.00)
They are key players in the health system	68 (10.0)	174 (25.7)	132 (19.5)	27 (4.0)	277 (40.9)	3.00 (3.00)
They are not competent enough to engage in patient care	18 (2.7)	52 (7.7)	172 (25.4)	63 (9.3)	373 (55.0)	5.00 (2.00)
I doubt the pharmacist level of clinical knowledge	26 (3.8)	46 (6.8)	162 (23.9)	62 (9.1)	382 (56.3)	5.00 (2.00)
They should just be at the pharmacy, not in the wards	30 (4.4)	46 (6.8)	159 (23.5)	74 (10.9)	369 (54.4)	5.00 (2.00)

SA, strongly agree; A, agree; N, neutral; D, disagree; SD, strongly disagree.

The median perception score of the survey participants was found to be significantly associated with age, experience in the current hospital, type of hospital, and hospital ownership (Table 3). For example, participants aged 41–50 years had a significantly higher median score than those aged 21–30 years (Median = 3.00, IQR = 4.00; vs Median = 2.00, IQR = 0.00; *p* = 0.006). Likewise, the perception of participants toward hospital pharmacists was significantly higher among those who had <1 year of experience in their current hospital compared with those with 5–8 years’ experience (Median = 2.00, IQR = 2.00; vs Median = 2.00, IQR = 1.00; *p* < 0.001), and working in a private hospital compared with public hospitals (Median = 2.00, IQR = 4.00; vs Median = 2.00, IQR = 2.00; *p* < 0.001).

**TABLE 3:** Median score association with demographics.

Variable	Median perception score (IQR)	*p*-value	Median expectation score (IQR)	*p*-value	Median experience score (IQR)	*p*-value
Gender						
Male	2.00 (2.00)	0.90	1.00 (1.00)	0.94	5.00 (2.00)	0.05
Female	2.00 (2.00)		1.00 (0.00)		5.00 (2.50)	
Age (years)						
21–30	2.00 (0.00)		1.00 (1.00)		2.50 (1.50)	
31–40	2.00 (2.00)		1.00 (0.00)		5.00 (2.00)	
41–50	3.00 (4.00)	0.006	1.00 (0.00)	<0.001	5.00 (0.00)	<0.001
>50	2.00 (2.50)		1.00 (0.00)		5.00 (0.00)	
Total-experience						
<1 year	2.00 (0.00)	0.20	2.00 (1.00)	<0.001	2.00 (0.50)	<0.001
1–5 years	2.00 (0.00)		1.00 (1.00)		2.50 (1.50)	
6–10 years	2.00 (1.75)		1.00 (1.00)		2.25 (1.00)	
>10 years	2.00 (3.75)		1.00 (0.00)		5.00 (0.00)	
Experience in current hospital						
<1 year	2.00 (0.00)	0.01	2.00 (1.00)	<0.001	2.00 (0.50)	<0.001
1–4 years	2.00 (0.00)		1.00 (1.00)		2.50 (1.50)	
5–8 years	2.00 (1.00)		1.00 (1.00)		2.25 (1.00)	
>8 years	2.00 (4.00)		1.00 (0.00)		5.00 (0.00)	
Education						
Bachelor such as MBBS	2.00 (1.00)		1.00 (1.00)		5.00 (2.50)	
Postgraduation	2.00 (2.00)	0.20	1.00 (0.00)	0.03	5.00 (1.00)	<0.001
Area of expertize						
Medicine	2.00 (2.00)		1.00 (0.00)		5.00 (0.00)	
Pediatric	2.00 (2.00)		1.00 (1.00)		5.00 (2.00)	
Surgery	2.00 (1.00)	<0.001	1.00 (0.00)	0.34	5.00 (2.38)	<0.001
Obstetrics and gynecology	2.00 (2.00)		1.00 (1.00)		3.25 (3.00)	
Others	2.00 (1.00)		1.00 (1.00)		3.00 (3.00)	
Hospital type						
Tertiary care	2.00 (2.00)		1.00 (0.00)		5.00 (2.00)	
Secondary care	2.00 (1.00)	0.07	1.00 (1.00)	0.97	5.00 (2.50)	0.04
Hospital ownership						
Public	2.00 (2.00)		1.00 (1.00)		5.00 (2.50)	
Private	2.00 (4.00)	0.01	1.00 (0.00)	0.20	5.00 (0.00)	<0.001

### Expectations of Physicians

Many physicians strongly agreed that pharmacists should attend patient care rounds to promptly respond to patient medication-related questions (*n* = 574, 84.7%) and to assist them in designing optimal drug therapy plans for patients (*n* = 545, 80.4%). More than two-quarters of physicians said pharmacists should review patients’ medications (*n* = 421, 62.1%) and prescriptions for appropriate dosage regimens, drug interaction, and allergies (*n* = 441, 65.0%). An appreciable number of physicians (*n* = 446, 65.8%) also strongly agreed with the role of pharmacists in prescribing cost-effective medicines and their monitoring, especially for antibiotics, to rationalize their use (*n* = 473, 69.8%). Similarly, 434 (64.0%) physicians sought to collaborate with pharmacists (Table 4).

**TABLE 4:** Expectations of physicians about pharmacists.

Question	Strongly agree; strongly disagree. *N* (%)					Median (IQR)
	SA	A	N	D	SD	
I expect the pharmacist to be on patient-care rounds to answer questions about patients’ medications	574 (84.7)	93 (13.7)	9 (1.3)	0 (0.0)	2 (0.3)	1.00 (0.00)
I expect pharmacists to assist me in designing drug therapy treatment plans for my patients	545 (80.4)	109 (16.1)	19 (2.8)	1 (0.1)	4 (0.6)	1.00 (0.00)
I expect pharmacists to educate my patients about the safe and appropriate use of their medications	485 (71.5)	137 (20.2)	44 (6.5)	4 (0.6)	8 (1.2)	1.00 (1.00)
I would be more confident if a clinical pharmacist was available on the wards to answer drug-related queries	448 (66.1)	127 (18.7)	77 (11.4)	4 (0.6)	22 (3.2)	1.00 (1.00)
I expect the pharmacist to review my patients’ medications for appropriateness (dose, indication, route, duration)	421 (62.1)	143 (21.1)	80 (11.8)	3 (0.4)	31 (4.6)	1.00 (1.00)
The pharmacist should check my prescription for potential drug-drug interactions, drug-disease interactions, and allergies	441 (65.0)	142 (20.9)	60 (8.8)	2 (0.3)	33 (4.9)	1.00 (1.00)
Pharmacists should participate in monitoring the rational use of drugs, especially antibiotics	446 (65.8)	149 (22.0)	48 (7.1)	1 (0.1)	34 (5.0)	1.00 (1.00)
I wish to collaborate with pharmacists for better patient care and treatment outcome	434 (64.0)	150 (22.1)	51 (7.5)	1 (0.1)	42 (6.2)	1.00 (1.00)
I expect the pharmacist to detect clinical problems with physicians’ prescriptions and advising when necessary	328 (48.4)	140 (20.6)	59 (8.7)	1 (0.1)	150 (22.1)	2.00 (2.00)
I expect the pharmacist to monitor patient response to drug therapy from the toxicity/side effects perspective	324 (47.8)	158 (23.3)	49 (7.2)	2 (0.3)	145 (21.4)	2.00 (2.00)
I expect pharmacists to help me in prescribing cost-effective medicines	473 (69.8)	131 (19.3)	28 (4.1)	2 (0.3)	44 (6.5)	1.00 (1.00)

SA, strongly agree; A, agree; N, neutral; D, disagree; SD, strongly disagree.

The expectations of physicians toward pharmacists were significantly higher among physicians with higher education such as a postgraduate vs only a graduate qualification (Median = 1.00, IQR = 1.00; vs Median = 1.00, IQR = 0.00; *p* < 0.001), experience <1 year vs. 5–8 years (Median = 2.00, IQR = 1.00; vs Median = 1.00, IQR = 1.00; *p* < 0.001) and aged 41–50 years vs 21–30 years (Median = 1.00, IQR = 0.00; vs Median = 1.00, IQR = 1.00; *p* < 0.001) as shown in Table 3.

### Experience of Physicians

Surprisingly, in view of their expectations, the majority of physicians strongly disagreed that pharmacists routinely inform them about cost-effective alternatives (*n* = 455, 67.1%) and potential issues in their prescriptions (*n* = 443, 65.3%). Very few physicians (*n* = 124, 18.3%) believed that pharmacists also counsel their patients about their medication’s judicial use. The role of pharmacists was currently viewed to be negligible by physicians in communicating medication-related issues of patients (*n* = 124, 18.3%). This compares with 232 (34.2%) of physicians agreeing that pharmacists involve in clarifying pharmacotherapy objectives (Table 5), and more than two-quarters of physicians (*n* = 368, 54.3%) considered the pharmacist as a trustworthy source of drug information showing contradictions in the responses.

**TABLE 5:** Experience of physicians about pharmacists.

Question	Strongly agree; strongly disagree. *N* (%)					Median (IQR)
	SA	A	N	D	SD	
Pharmacists routinely inform me if they notice potential problems in my prescriptions	21 (3.1)	43 (6.3)	83 (12.2)	76 (11.2)	455 (67.1)	5.00 (1.00)
Pharmacists routinely inform me about more cost-effective alternatives	42 (6.2)	41 (6.0)	77 (11.4)	75 (11.1)	443 (65.3)	5.00 (1.00)
In my experience, pharmacists are a trustworthy source of general drug information	217 (32.0)	151 (22.3)	25 (3.7)	24 (3.5)	261 (38.5)	2.00 (4.00)
Pharmacists routinely advise my patients as to the safe and appropriate use of their medications	58 (8.6)	66 (9.7)	81 (11.9)	66 (9.7)	407 (60.0)	5.00 (2.00)
I create a relationship with pharmacists when the pharmacist tries to adjust my patients’ medication	78 (11.5)	156 (23.0)	27 (4.0)	22 (3.2)	395 (58.3)	5.00 (3.00)
Pharmacists routinely let me know that my patients have encountered some problems with their medications	47 (6.9)	77 (11.4)	73 (10.8)	64 (9.4)	417 (61.5)	5.00 (2.00)
In my experience, pharmacists tend to take personal responsibility for managing any drug-related problems	180 (26.5)	163 (24.0)	17 (2.5)	17 (2.5)	301 (44.4)	2.00 (4.00)
Pharmacists regularly inquire me to clarify for them the pharmacotherapy objectives that I have in my mind	85 (12.5)	147 (21.7)	25 (3.7)	22 (3.2)	399 (58.8)	5.00 (3.00)

SA, strongly agree; A, agree; N, neutral; D, disagree; SD, strongly disagree.

A significant association was found between the median experience score of physicians with gender, age, total experience within hospitals, experience in the current organization, education, type of hospital, ownership of the hospital, area of expertize, and the current position of physicians (Table 3). For example, the median experience score was significantly higher among physicians aged 41–50 years than those aged between 21–30 years (Median = 5.00, IQR = 0.00; vs Median = 2.50, IQR = 1.50; *p* < 0.001). Physicians with greater experience (>5 years) had a significantly higher median score toward pharmacists than physicians with <1 year of experience (Median = 5.00, IQR = 0.00; vs Median = 2.00, IQR = 0.50; *p* < 0.001). Similarly, the median score of physicians working in internal medicine was significantly associated with those with other specialities such as Obstetrics and Gynecology (Median = 5.00, IQR = 0.00; vs Median = 3.25, IQR = 3.00; *p* < 0.001).

## Discussion

This study provides an empirical account on the perceptions, expectations, and experience of Pakistani physicians working in different hospital settings toward hospital pharmacists. The results show that physicians have a polarized view of the patient care-related roles of pharmacists, but portray a higher level of expectation, especially in services that support their role in the healthcare system.

The surveyed physicians perceived a significant role of pharmacists in patient counseling, recommending dosage adjustments, detecting and managing adverse events, and solving drug-related issues. Likewise, physicians recognized the role of pharmacists as sources of reliable drug information who can share the responsibility with physicians in improving patient outcomes. This is perhaps not surprising as pharmaceutical companies have been a key source of drug information in the past in Pakistan (Riaz et al., 2015). Similar findings have also been highlighted in the literature (Almazrou et al., 2019; Kabba et al., 2020; Said et al., 2020). Encouragingly, a significant number of physicians advocated the involvement of pharmacists in wards, which could help them in getting prompt feedback on drug-related issues and pharmacotherapy plans for patients.

Several studies have shown that optimal patient outcomes can be achieved through effective collaboration among healthcare professionals (Farrell et al., 2010; Curran et al., 2019). However, in our study, inadequate interaction was reported by most of the physicians with pharmacists despite the perceived significant role of pharmacists in improving the pharmaceutical care of patients. This was illustrated by the fact that most of the inquiries made by physicians were related to drug availability. This limited acceptability of pharmacists’ engagement in performing greater clinical roles in hospitals in Pakistan could be due to insufficient knowledge and poor perception among physicians. Similar results have been reported from developing countries such as Iran, Ethiopia, and Jordan (Tahaineh et al., 2009; Alipour et al., 2018; Kabba et al., 2020). Good clinical knowledge is essential for pharmacists alongside appropriate pharmaceutical knowledge to help guide treatment approaches and address concerns. (Zaidan et al., 2011; Alipour et al., 2018). However, there is currently a lack of adequate numbers of pharmacists in different hospital settings in Pakistan. Presently, there is only one pharmacist for 1,200 beds in most of the hospitals in Pakistan, which is considerably less than one pharmacist per 50 beds standard established by the WHO to fully appreciate their role and value in providing pharmaceutical care (News, 2016). The Pharm D curriculum developed by the Pharmacy Council of Pakistan (PCP) clearly demonstrates that pharmacists should be provided training regarding the signs and symptoms of patients with different diseases, including a physical examination. However, this assumes time to undertake such tasks. This needs to be addressed going forward.

Encouragingly, a high level of expectation on the pharmacist’s role in patient care was perceived by most of the physicians. This was illustrated by the response of physicians to patient medication issues and reflected the high expectation of physicians regarding the role of pharmacists in hospitals (Li et al., 2014; Sabry and Farid, 2014). However, the collaboration between physicians and pharmacists in improving patient care and outcomes is currently lacking in our study owing to the perceived insufficient interaction between pharmacists and physicians. Physicians have considered this suboptimal interaction as a barrier in effective physician-pharmacist collaboration (Alkhateeb et al., 2009), which also needs to be addressed going forward.

Another key role for pharmacists where there are workforce and other issues is on immunization programs, which are routinely undertaken in hospitals in Pakistan. The role of pharmacists in helping with immunization is well established in numerous developed countries including the United Kingdom and United States; however, this practice is still challenging in developing countries (Poudel et al., 2019; Yemeke et al., 2020). In concordance with previous studies (Moore et al., 2014; Kabba et al., 2020), the participants of our study currently showed skepticism toward the role of pharmacist-led immunization. This may be due to the limited awareness among physicians of the rapidly evolving role of pharmacists. Secondly, physicians do not view vaccines as medicines (Wilson et al., 2015). This also needs to be addressed going forward especially post the current pandemic.

Given the limited experience of physicians with certain activities of pharmacists, the expectations of physicians with new patient-oriented roles were promising as seen in other studies (Zaidan et al., 2011).

We are aware that our study has several limitations. Firstly, the study was conducted in only one province of Pakistan and the findings may limit its generalizability to the whole country. However, this is the largest province in Pakistan. Second, a convenience sampling technique was employed, which may offer selection bias. However, this is a limitation of all studies involving this approach. Thirdly, this study only investigated the view of physicians regarding pharmacist roles. Studies with other healthcare professionals such as nurses and microbiologists are needed to enhance the strength of the message. Despite the above limitations, we believe our findings are robust providing direction for the future.

## Conclusion

This study shows that physicians were interested in the emerging roles of pharmacists and contemplated them as a trustworthy source of knowledge about drug information. Nevertheless, the expectations and perceptions of physicians regarding pharmacists’ roles mismatch with their experiences and activities to date with the latter owing to the limited interprofessional relationship. This is exacerbated by currently limited numbers of hospital pharmacists and their multiple roles and needs addressed to appreciably enhance the role of hospital pharmacists providing pharmaceutical care in Pakistan in the future.

### Recommendations

Serious efforts to increase physicians’ awareness about the importance of interprofessional collaboration are urgently needed. Clinical rotations of pharmacy students with physicians should be integrated to direct patient care. It is imperative to include courses pointing to physician-pharmacist collaboration into the curriculum, which will help to improve the quality of patient care. Besides, the Ministry of Health (MoH) of Pakistan should increase the number of pharmacists in hospitals by following the WHO’s guidelines.

## Data Availability

The raw data supporting the conclusions of this article will be made available by the authors, without undue reservation.
