# Supplementary material for: Perceptions, Expectations, and Experience of Physicians About Pharmacists and Pharmaceutical Care Services in Pakistan: Findings and Implications
Source: Front Pharmacol. 2021 May 14;12:650137. doi: 10.3389/fphar.2021.650137 (PMC8160509; doi:10.3389/fphar.2021.650137)
Supplement: Supplementary file 1 [file DataSheet1.docx]

**Perception, expectation, and experience of physicians about pharmacists and pharmacy services in Punjab, Pakistan**

**Section A. Sociodemographic characteristics**

1. **Age (years).**

☐ 21-30yrs ☐ 31-40yrs ☐ 41-50yrs ☐ >50yrs

**2. Gender**

☐ Male ☐ Female

**3. How many years have you been practicing medicine**

☐ Less than 1years ☐ 1-5 yrs. ☐ 6-10yrs ☐ More than 10yrs

**4. How many years have you been working in this hospital?**

☐ Less than 6 month ☐ 1-4years ☐ 5-8 years ☐ More than 8years

**5. About how many prescriptions do you prescribe weekly?**

☐1-20 ☐21-50 ☐51-100 ☐More than 100

**6. Education level**

☐ Bachelor’s degree in medicine. ☐ Postgraduate degree/diploma/fellowship.

**7. Current area of practice**

☐Internal medicine. ☐Pediatric. ☐ Surgery. ☐Obstetrics& Gynecology ☐Others

…

**8. Type of hospital**

☐ Tertiary hospital. ☐ Secondary hospital

**Section B. Physician-pharmacists interaction**

**1. Physician-pharmacist interaction**

☐Never or rarely ☐1–2 times every month ☐3–6 times every month ☐6–10 times every month ☐>10 times every month

**2. What are the reasons for interaction with pharmacists?**

a) Drug-availability enquiries ☐ Yes ☐ No

b) Side effects enquiries ☐ Yes ☐ No

c) Drug-alternative enquiries ☐ Yes ☐ No

d) Drug-dosage enquiries ☐ Yes ☐ No

e) Drug-interaction enquiries ☐ Yes ☐ No

**Section C. Perception of physicians about pharmacists**

Please tick one: *strongly agree, agree, neutral, disagree, or strongly disagree* for each of the statements below.

|  | **Items** | **Strongly agree** | **Agree** | **Neutral** | **Disagree** | **Strongly disagree** |
| --- | --- | --- | --- | --- | --- | --- |
| 1 | They provide evidence-based drug information |  |  |  |  |  |
| 2 | They solve drug-related problems |  |  |  |  |  |
| 3 | They contribute to improving patient care |  |  |  |  |  |
| 4 | They provide Patient counselling on appropriate drug use. |  |  |  |  |  |
| 5 | They are accessible in the hospital |  |  |  |  |  |
| 6 | They play an important role in improving patient outcomes |  |  |  |  |  |
| 7 | They recommend dosage adjustments |  |  |  |  |  |
| 8 | They Identify and help resolve adverse drug reactions |  |  |  |  |  |
| 9 | They take part in patient-care rounds |  |  |  |  |  |
| 10 | They participate in research programs |  |  |  |  |  |
| 11 | They participate in vaccination programs |  |  |  |  |  |
| 12 | They are key players in the health system. |  |  |  |  |  |
| 13 | They are not competent enough to engage in patient care. |  |  |  |  |  |
| 14 | I doubt the pharmacist level of clinical knowledge |  |  |  |  |  |
| 15 | They should just be at the pharmacy, not in the wards. |  |  |  |  |  |

**Section D: Doctors Expectations about pharmacists**

Please tick one: *strongly agree, agree, neutral, disagree, or strongly disagree* for each of the statements below.

|  | **Items** | **Strongly agree** | **Agree** | **Neutral** | **Disagree** | **Strongly disagree** |
| --- | --- | --- | --- | --- | --- | --- |
| 1 | I expect the pharmacist to be on patient-care rounds to answer questions about patients’ medications |  |  |  |  |  |
| 2 | I expect pharmacists to assist me in designing drug therapy treatment plans for my patients. |  |  |  |  |  |
| 3 | I expect Pharmacists to educate my patients about the safe and appropriate use of their medications |  |  |  |  |  |
| 4 | I would be more confident if a clinical pharmacist was available on the wards to answer drug information related quarries |  |  |  |  |  |
| 5 | I expect the pharmacist to review my patients’ medications for appropriateness (dose, indication, route, duration) |  |  |  |  |  |
| 6 | The pharmacist should check my prescription for potential drug interactions, drug-disease interactions, and allergies |  |  |  |  |  |
| 7 | Pharmacists should participate in monitoring the rational use of drugs, especially antibiotics. |  |  |  |  |  |
| 8 | I wish to collaborate with pharmacists for better patient care and treatment outcome. |  |  |  |  |  |
| 9 | I expect the pharmacist to detect clinical problems with physicians’ prescriptions and advising when necessary |  |  |  |  |  |
| 10 | I expect the pharmacist to monitor patient response to drug therapy from the toxicity/side effects perspective |  |  |  |  |  |
| 11 | I expect pharmacists to help me in prescribing cost-effective medicines |  |  |  |  |  |

**Section E: Experience of physicians with pharmacists**

(Please tick from strongly agree to strongly disagree in the below mentioned questions)

|  | **Items** | **Strongly agree** | **Agree** | **Neutral** | **Disagree** | **Strongly disagree** |
| --- | --- | --- | --- | --- | --- | --- |
| 1 | Pharmacists routinely inform me if they notice potential problems in my prescriptions |  |  |  |  |  |
| 2 | Pharmacists routinely inform me about more cost-effective alternatives |  |  |  |  |  |
| 3 | In my experience, pharmacists are a trustworthy source of general drug information |  |  |  |  |  |
| 4 | Pharmacists routinely advise my patients as to the safe and appropriate use of their medications |  |  |  |  |  |
| 5 | I create a relationship with pharmacists when the pharmacist tries to adjust my patients’ medication |  |  |  |  |  |
| 6 | Pharmacists routinely let me know that my patients have encountered some problems with their medications |  |  |  |  |  |
| 7 | In my experience, pharmacists tend to take personal responsibility for managing any drug-related problems |  |  |  |  |  |
| 8 | Pharmacists regularly inquire me to clarify for them the pharmacotherapy objectives that I have in my mind |  |  |  |  |  |

**Thank you very much for your time and willingness to take part in this survey**
